# Validating an Online Portuguese Battery to Measure Working Memory Capacity in a Sample of Portuguese and Brazilian Participants

**DOI:** 10.5334/joc.489

**Published:** 2026-02-12

**Authors:** Nuno Gaspar, Alessandra S. Souza, Márcia Maria Peruzzi Elia da Mota, Carlos Eduardo Nórte

**Affiliations:** 1 Faculty of Psychology and Education Sciences, University of Porto, PT; 2 Center for Psychology, Faculty of Psychology and Education Sciences, University of Porto, PT; 3 Salgado de Oliveira University, Niterói, BR; 4 Institute of Psychology, State University of Rio de Janeiro, BR

**Keywords:** working memory capacity, online tasks, Portuguese language, Portugal, Brazil

## Abstract

This article introduces a computerized set of online tasks suitable for measuring working memory capacity among European Portuguese and Brazilian Portuguese speakers (the oWMC-PT battery). The battery comprises three working memory measures: the reading span, the symmetry span, and the forward digit span. The tasks can be completed online at the participant’s own devices in approximately 30 min. The oWMC-PT was specifically designed for use with Portuguese and Brazilian populations but can be readily adapted for any language (and we provide an English version in our repository). The three tested tasks had acceptable reliability and correlated moderately, forming a single latent factor reflecting working memory capacity. We observed configural and metric invariance, but not scalar invariance, between the two tested samples of speakers of European Portuguese (N = 195) and Brazilian Portuguese (N = 154). This indicates that tasks load similarly to the latent factor in both samples, but latent scores between samples cannot be directly compared. The oWMC-PT task can serve as a versatile tool to assess working memory capacity in online samples – particularly in the Portuguese-speaking world, but potentially also in other languages. The battery is accessible at: https://osf.io/ubcez.

Working memory (WM) is considered a central construct in human cognition, being implicated in learning, problem-solving, reasoning, and other high cognitive functions relevant to clinical, developmental, and educational fields (Chai et al., 2018). WM can be understood as a system that temporarily stores and manipulates information making it accessible for use in other cognitive activities (Baddeley, 1986, 2012; Baddeley & Hitch, 1974; Cowan, 2017; Engle, 2002; Oberauer, 2009). The capacity of WM is limited, and individual differences in WM capacity are related to several other abilities such as intelligence (Ackerman et al., 2005; Kyllonen & Christal, 1990; Rey-Mermet et al., 2019; Süß et al., 2002), reading comprehension (Peng et al., 2018), mathematics (Friso-van den Bos et al., 2013), and academic achievement in general (Alloway & Alloway, 2010). It is, therefore, extremely important to measure WM capacity accurately.

Several authors (Barreyro et al., 2019; Lewandowsky et al., 2010; Ma et al., 2017; Monteiro et al., 2024; Oswald et al., 2015; Stone & Towse, 2015) have developed and made publicly available task batteries that can be used to measure WM at the latent level – i.e., by extracting a factor reflecting individual variation in this construct unconfounded by task-specific variance (Oberauer, 2005; Wittmann, 1988). At present, we are aware of only one battery recently made available in Portuguese (Monteiro et al., 2024). Nevertheless, the tasks available in this battery are not meant to be completed online and were not validated to be used both in Portugal and Brazil. The goal of the present study was to develop, test, and make available a short (ca. 30 min completion time) Portuguese battery to measure WM capacity (oWMC-PT for short) that uses a freely available platform (lab.js; Henninger et al., 2022), with tasks that can be completed online or offline, and that can be simultaneously applied in Portugal and Brazil, and potentially other Portuguese speaking countries as well. The battery can be also translated and validated in other languages and to facilitate this process we provide an English version in our repository.

## How to Measure WM Capacity?

Measures of WM capacity are often divided into assessing the memory maintenance component of the system (also known as passive measures) and assessing both maintenance and information-processing functions (also known as active measures). One of the most common measures of the memory maintenance component of WM is the so-called simple span tasks, such as *word span* or *digit span*. This type of task requires serial memory for a sequence of words or digits, usually presented at a rate of one per second, with subsequent ordered recall of the whole sequence. Simple span tasks, and in particular digit span, have been used as a measure of immediate memory performance for decades. They figure in well-established tests of verbal intelligence (e.g., Wechsler, 2012), and are common in the neuropsychological literature (Ramsay & Reynolds, 1995). Although considered to have less predictive variance for high cognition than other more complex WM measures, a recent meta-analysis indicates that digit span is a good predictor of performance in reasoning tests (Gignac & Weiss, 2015). Accordingly, we included the digit span task^1^ as an indicator of the maintenance component of WM in our battery.

Although simple span tasks can be considered as a measure of storage capacity only, other paradigms have been developed to measure both the memory and processing functions of the WM system. In this regard, complex span tasks are the most frequently used paradigm for assessing WM (Conway et al., 2005; Redick et al., 2012). Complex span tasks require participants to memorize a series of stimuli presented in succession, while concurrently processing other information. For example, participants may be required to read a sentence or solve a mathematical operation, while memorizing letters, words, numbers, or even visuospatial information. The most common types of complex span tasks are: operation span (Turner & Engle, 1989; Unsworth et al., 2005), reading span (Daneman & Carpenter, 1980), and symmetry span (Kane et al., 2004). Complex span tasks usually demonstrate excellent psychometric properties: they have good internal consistency, stability over time, and convergent and criterion validity (Redick et al., 2012). Accordingly, in our battery, we included two complex span tasks that differ in the memory materials: (1) reading span – in which participants had to read and evaluate the meaning of a sentence, while memorizing its last word; and (2) symmetry span – in which participants judged the symmetry of a matrix along its vertical axis, and then memorized a location on a grid. We selected a verbal and a visual complex span task – in addition to the digit span that includes numerical materials – to make sure our assessment of WM capacity reflected a domain-general component (Kane et al., 2004; Oberauer et al., 2000; Wilhelm et al., 2013).

## Why an Additional Task Battery?

Although several task batteries are already available for assessing WM performance, language, software, and location constraints still represent barriers to several researchers. With regards to language barriers, up to 2024, there was no openly available battery to assess WM for Portuguese speakers. Monteiro et al. (2024) provided access to the first battery in this language that we are aware of. Yet, this study developed the tasks to be used only in Portugal, which represents only a relatively small fraction of the Portuguese-speaking population. Achieving equivalent language versions is relevant when comparing national or cultural groups. Portugal and Brazil together have a population of 213 million people. Since 2009, an orthographic agreement has been established between the two countries, standardizing the written use of Portuguese. Yet, language use is not always comparable across these countries; hence, in the present battery, we selected materials and instructions that were similarly understandable in both countries, thereby making it possible to apply the same instrument in Portugal and Brazil.

With regards to software and location barriers, so far, most studies have made available task batteries programmed in proprietary software (e.g., Matlab or E-prime) which require an expensive subscription that may not be accessible to all researchers, particularly in countries with fewer resources as in Brazil or other Portuguese-speaking countries (e.g., Angola). Additionally, most batteries are assumed to be completed in a laboratory setting, under close supervision of the experimenter. After the COVID-19 pandemic, we have seen a rise in interest in making online, distant assessments available, which increases the flexibility in data collection. Our battery bridges all these three gaps: it can be used in Portugal and Brazil, it is programmed in free software, and it can be completed online or offline while also being relatively short (completion time between 30 to 40 min). The full task battery is available in a public repository together with a guide on how researchers can use and adapt it to their specific purposes.

## The Present Study

This study was motivated by the following objectives: (1) To develop a computerized WM assessment battery in Portuguese that can be used both in Brazil and Portugal – and potentially other Portuguese-speaking countries as well; (2) To apply this battery remotely to participants in both countries – participants received a link to the study and completed the assessment online, on their own devices, without supervision; (3) To examine if the tasks capture common variance in memory performance that can establish a WM capacity factor; and (4) To assess if the obtained factor structure is comparable across countries.

## Method

### Participants

The participants were 351 volunteers between the ages of 15 and 65 (*M* = 27.95 years, *SD* = 12.37), from Portugal (N = 195; 55.6%), Brazil (N = 154; 43.9%), and other unspecified countries (N = 2; 0.57%). We excluded the two participants who indicated that they were not from Portugal and Brazil, leaving a total of N = 349. The study was conducted in the second semester of 2021. This study was approved by the Ethics Committee of the Faculty of Psychology and Education Sciences of the University of Porto (Ref.ª 2021/07-05b). Before recruitment, data protection compliance with applicable laws was ascertained by the Data Protection Unit of the University of Porto.

In Portugal, the study was disseminated via an email, issued by the University of Porto’s central services, inviting for a 40-minute online study. The email was sent to all the students (35022) and staff of the University of Porto community (7309). In Brazil, the study was disseminated among Psychology university students by faculty (authors CEN and MMM), the participants conducted the experiment at home or in a classroom setting. However, in both cases, task completion was autonomous, and the instructions were the same. Participants were invited through posters distributed at the university and also in the classroom. In both countries, participation was voluntary: participants read and accepted an online informed consent form. No financial or other compensation was given. The data of all participants who completed all three tasks were retained for analysis.

Table 1 presents the demographic information of the two tested samples. The Brazilian sample is younger, on average, than the Portuguese sample. Most of the participants were women in both countries. As this study was conducted while the Covid-19 pandemic was ongoing (second semester of 2021), we also asked participants to disclose if they had been exposed to Covid. More participants from Brazil (42.9%) had Covid compared to Portugal (8.7%). Given that the study in Portugal was advertised to all university members, whereas in Brazil it was conducted with Psychology students, the sample in Portugal included more participants with a higher level of education (master and doctoral degrees; 38.5%) than in Brazil (3.9%). Given these differences in terms of exposition to Covid-19 and education level, we considered the impact of these two variables on performance in our tasks.

**Table 1:** Break Down of the Demographic Variables for the Two Tested Samples.


VARIABLE		PORTUGAL (N = 195)	BRAZIL (N = 154)

Age	Mean	31.85	23.01

	SD	13.39	8.75

	Range	17–65	17–61

Gender	Woman	131 (67.2%)	114 (74%)

	Man	61 (31.3%)	35 (22.7%)

	Rather not say	3 (1.5%)	5 (3.2%)

Covid-19	Yes	17 (8.7%)	66 (42.9%)

	No	178 (91.3%)	88 (57.1%)

Education	No education	0	1 (0.6%)

	9th class	1 (0.5%)	0

	High school	52 (26.7%)	63 (40.9%)

	Bachelor degree	67 (34.4%)	84 (54.5%)

	Master degree	44 (22.6%)	4 (2.6%)

	Doctoral degree	31 (15.9%)	2 (1.3%)

Mother Educ.	No education	1 (0.5%)	1 (0.6%)

	Elementary school	32 (16.4%)	5 (3.2%)

	6th Grade	17 (8.7%)	7 (4.5%)

	Middle School	21 (10.8%)	2 (1.3%)

	High-school	42 (21.53%)	39 (25.3%)

	Bachelor degree	62 (31.8%)	61 (39.6%)

	Master degree	13 (6.7%)	27 (17.5%)

	Doctoral degree	7 (3.6%)	12 (7.8%)


### Transparency and Openness

All experimental materials (stimuli and tasks), as well as the data and analysis code, are fully available at the Open Science Framework at https://osf.io/ubcez (Souza & Gaspar, 2024). The study was not pre-registered.

### Materials & Procedure

We implemented three working memory span tasks that were completed in this order: The reading span task (Daneman and Carpenter, 1980), the symmetry span task (Kane et al., 2004), and the digit span task. All participants completed the three tasks in the same order, because we were interested in measuring individual-level performance and not task-order effects. As individual-level performance parameters may be compromised by task-order effects, it is common in individual difference studies to fix task order (see Wilhelm et al., 2013). We presented the complex span tasks first, as they are more demanding, and task performance could be more impaired by fatigue. We also tried to vary content domain by inserting the visuospatial task in between the two more verbal tasks. Computerized versions of each task, described below, were developed using lab.js (Henninger et al., 2022).

The Portuguese participants completed the tasks online on their own devices. The Brazilian sample was collected during in-person classes, using the same computerized tasks, or the links were sent for completion at home. In both cases, instructions were the same in order to maintain similar conditions. The tasks were designed to maximize completion rates and motivation. Participants were not allowed to go back to previous phases of any of the tasks. Performance feedback, in the form of percentage of attained success, was presented at the end of each task.

#### Reading Span

**Figure 1:**
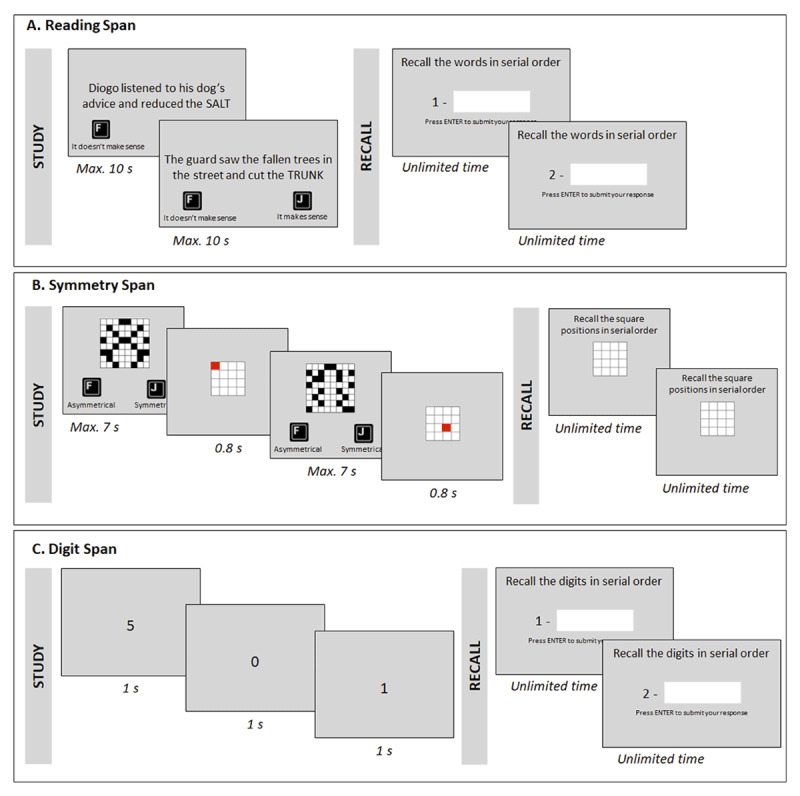
Illustration of the Flow of Events in the Three Working Memory Tasks: A. Reading Span, B. Symmetry Span, and C. Digit Span.

This task is based on a Portuguese adaptation of the Daneman and Carpenter (1980) task, developed by Gaspar and Pinto (2001), which was modified to create a computerized task suitable for Portuguese and Brazilian participants. Figure 1A illustrates the flow of events in this task.

The computerized version of the reading span task involves: a) reading each sentence; b) assessing the meaning of the sentence (by pressing the keyboard keys ‘F’ for meaningless or ‘J’ for meaningful); and c) memorizing the last word of each sentence (printed in capitalized font). Participants were given 10 seconds to read and classify each sentence, with the progress in the experiment being automatically paced. During recall, participants were instructed to enter using the keyboard the memorized words in the order they were presented.

The task began with three practice trials (with extensions 2, 4, and 3), followed by 15 experimental trials. Each trial comprised 2 to 6 sentences (each level being presented three times), randomly intermixed. One random trial order was generated and remained consistent for all participants.

Each sentence of the task underwent analysis by both a Portuguese and a Brazilian researcher to ensure that semantic, syntactic, and/or other sentence properties were similar for Portuguese and Brazilian samples. A total of 69 sentences (34 meaningful and 35 meaningless) were created following these guidelines. The list of sentences used in this task can be found in our OSF project.

#### Symmetry Span

The symmetry span task, developed by Kane et al (2004), requires making judgments about the vertical axis symmetry of 8 × 8 black-and-white matrices, followed by memorizing the position of a red square in a 4 × 4 matrix (see Figure 1B). Each trial begins with the presentation of an 8 × 8 matrix, to be classified as symmetric or asymmetric (by pressing the F and J keys, respectively) within 7s. Immediately after, a red square is presented at a to be remembered position in a 4 × 4 matrix. This sequence is repeated between 2 and 6 times per trial. At recall, an empty 4 × 4 matrix is presented. Participants are prompted to click on the position of the memorized red squares in their order of presentation.

Participants completed 3 practice trials (with extensions 2, 3, and 4) and 15 experimental trials with varying extensions, ranging from 2 to 6 red square positions to be recalled (3 trials per level), randomly intermixed. One random trial order was generated and remained consistent for all participants.

#### Digit Span

This classical task involves the visual presentation of sequences of single digits, from 0 to 9, at a pace of one digit per second (see Figure 1C). After the presentation of the digits sequence, participants were prompted to recall the digits in the order they were presented. The sequences could have an extension ranging from 3 to 10 digits. Within one trial sequence, the digits were never repeated.

Participants completed 3 practice trials (extensions 3, 4, and 6), and 24 experimental trials with sequence lengths between 3 and 10 (three repetitions of each level), which were randomly intermixed. A random trial order was generated and remained constant for all participants.

### Data Analysis

Performance was measured by the proportion of correct responses in each task component (memory and processing). Responses in the practice trials were excluded from the analyses. For each task, we computed a memory score by taking the proportion of to-be-remembered items recalled in the correct serial position across all trials, regardless of whether the entire sequence was recalled correctly in a trial and sequence length in each trial (i.e., the partial credit load scoring; Conway et al., 2005). We also checked performance in the processing task for completeness, but we retained all participants regardless of accuracy in the processing task.

We analyzed the factorial structure of the battery by submitting the scores from the three tasks to a multigroup confirmatory factor analysis (CFA) using the package *lavaan* (Rosseel, 2012 version 0.6.16) implemented in R (R core team, 2017). We tested for measurement invariance across samples by incrementally increasing the constraints of nested models. First, we allowed different intercepts and loadings between the tasks and the WM latent factor for each sample (*configural* invariance). Configural invariance tests whether the tasks measure the same construct across samples. Next, we constrained the task loadings to be the same between samples (*metric* invariance). Metric invariance indicates whether the strength of the relation between the indicators (i.e., the tasks) and the latent factor is the same between the samples. Finally, we constrained the loadings and intercepts to be the same between samples (*scalar* invariance). Scalar invariance indicates whether factor means can be compared across samples (i.e., a latent factor loading of 0.5 represents the same thing in both groups). To compare model fit between these three sets of nested models, we assessed the following indices: The chi-square goodness-of fit statistic (χ2), the root-mean-square error of approximation (RMSEA), the Bentler’s comparative fit index (CFI), and the Bayesian Information Criterion (BIC). For χ2, a small, non-significant value indicates a good fit. RSMEA smaller than 0.06 indicates a good fit, and of 0.08 or less, a reasonable fit. For the CFI, values higher than 0.95 indicate a good fit, and values between 0.90 and 0.95 indicate an adequate fit. Lower values of BIC indicate better model fit. We assessed changes in these indices across models and relied on the significance test for the difference in the chi-square between models. The cutoff criteria were based on Hu and Bentler (1999).

## Results

Table 2 presents the performance scores in each task for the Portuguese and Brazilian samples. Overall, the Portuguese sample obtained larger scores compared to the Brazilian sample in all tasks: reading span, *t*(324.35) = 1.994, *p* = 0.047, *d* = 0.22, symmetry span, *t*(329.82), *p* < .001, *d* = 0.56, and digit span, *t*(300.66) = 3.46, *p* = 0.0006, *d* = 0.38. Likewise, when considering performance in the processing task during complex span, the Portuguese sample outperformed the Brazilian one: reading processing, *t*(332.96) = 7.17, *p* < .001, *d* = 0.77, symmetry processing, *t*(257.42) = 5.07, *p* < .001, *d* = 0.57. There was a positive correlation between performance in the memory task and its processing component: Reading span, Portuguese *r*(193) = 0.19, *p* = .009, Brazilian *r*(152) = 0.16, *p* = .048; and Symmetry span, Portuguese *r*(193) = 0.30, *p* < .001, Brazilian *r*(150) = 0.47, *p* < .001. This indicates that people who performed well in the processing task also performed well in the memory task. This goes against the idea that people sacrifice performance on the processing task to achieve better memory scores (which would lead to a negative correlation). Lastly, all scores derived from the memory tasks had good reliability (Cronbach’s alpha above 0.8) for both samples. This indicates that the task items have good internal consistency.

**Table 2:** Descriptive Statistics and Reliability (Cronbach’s Alpha) of the Task Scores for each Sample.


MEASURE	SAMPLE	MEAN	SD	RANGE	SKEW	KURTOSIS	α

***Reading Span***							

memory	Portuguese	0.69	0.18	0.00–0.98	–1.04	1.44	0.86

	Brazilian	0.65	0.19	0.00–0.98	–1.02	1.82	0.87

processing	Portuguese	0.79	0.16	0.07–0.93	–3.05	9.85	

	Brazilian	0.67	0.15	0.00–0.87	–1.97	4.97	

***Symmetry Span***							

memory	Portuguese	0.60	0.20	0.10–0.98	–0.21	–0.82	0.87

	Brazilian	0.49	0.20	0.08–0.92	0.04	–0.75	0.87

processing	Portuguese	0.93	0.08	0.51–1.00	–2.56	7.91	

	Brazilian	0.87	0.12	0.49–1.00	–1.67	2.43	

***Digit Span***							

memory	Portuguese	0.63	0.12	0.32–0.94	0.02	0.00	0.83

	Brazilian	0.59	0.14	0.10–0.91	–0.27	0.59	0.86


*Note*. Scores were computed based on the accuracy in each task component (memory and processing).

We considered whether the difference in exposure to Covid-19 and in education level (i.e., participants reporting having a master or doctoral degree vs. not) explained differences in performance between the samples of the two countries. For each dependent variable, we conducted ANOVAs including country, Covid-19, and education level as factors. The results are presented in the Online Supplementary Materials. Overall, in none of the analyses, exposition to Covid-19 or education level had a significant main effect. There was only one analysis (memory scores from Symmetry Span) in which the three-way interaction between country, Covid-19 and education level was significant. This was due to performance of the Brazilian sub-sample with a higher education and no Covid exposition being higher than of the corresponding Portuguese sub-sample, whereas for all other subgroups, the Portuguese sample outperformed the Brazilian one.

Next, we computed the correlations between the memory scores in the three tasks (see Table 3). Scores in all three tasks were positively correlated for each sample, as it would be expected from all tasks measuring the same underlying construct, namely, WM capacity.

**Table 3:** Correlations Across Tasks per Sample. Values Below and Above the Diagonal Represent the Portuguese and the Brazilian Sample, Respectively.


MEASURE	READING SPAN	SYMMETRY SPAN	DIGIT SPAN

Reading Span		0.259	0.337

Symmetry Span	0.417		0.318

Digit Span	0.463	0.409	


**Figure 2:**
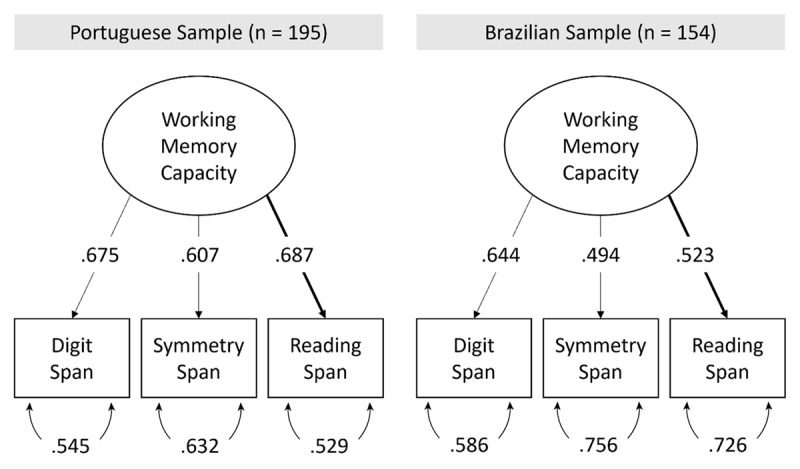
One-Factor Model of Working Memory Capacity with Standardized Loadings for the two Samples.

Finally, we submitted the memory scores to a CFA model that assumes that all three tasks load on a single latent factor representing WM capacity. We tested whether this model was a good representation of the relation between tasks in the two samples (configural invariance). Figure 2 illustrates the one-factor model fitted to the data testing for configural invariance between samples. We also assessed whether the tasks loaded similarly in the latent factor (metric invariance) and latent scores were equivalent (scalar invariance) between samples. Table 4 presents the fit indices of the three sets of nested models. Model comparison indicated that configural and metric invariance were established between the two samples, but not scalar invariance. The model with scalar invariance had a significant decrease in fit as indicated by a significant change in the chi-square, RMSEA, and CFI indices. Hence the three tasks can be used to assess WM capacity between countries, but factor scores cannot be directly compared across samples. We also evaluated whether these results hold when we retained in the sample only the participants that had, at maximum, a bachelor degree (i.e., excluding participants with a master or doctor degree which were more numerous in Portugal). The Online Supplementary Materials present the results of this analysis. The results remain unchanged, indicating that differences in education level in the two samples cannot account for the lack of scalar invariance between samples.

**Table 4:** Fit Indices for the Multigroup CFA testing for Configural, Metric and Scalar Invariance between the Portuguese and Brazilian Samples.


FIT INDICES	MODEL TEST OF INVARIANCE		

	CONFIGURAL	METRIC	SCALAR

χ^2^	0.000	1.156	8.975

Df	0	2	4

Δχ^2^		1.156	7.819

p-value (Δ χ^2^)		.561	.020*

CFI	1.000	1.000	0.962

Δ CFI		0.0	0.038

RMSEA	0.000	0.000	0.084

Δ RMSEA		0.0	0.084

BIC	–818.03	–828.58	–832.47


*Note*. Df = degrees of freedom. * Significant decrease in model fit compared to the configural model.

## Discussion

The goal of this study was to test the oWMC-PT battery by collecting data from Portuguese and Brazilian samples. The task scores revealed good internal consistency (Cronbach’s alpha > 0.8) and moderate correlations between each other, forming a single latent factor that revealed configural and metric invariance between the two tested samples. These results indicate that the oWMC-PT can be a useful tool to measure WM capacity in Portuguese speaking participants.

### Task Correlations

We implemented tasks using diverse sets of stimuli to assess WM capacity. The included tasks used verbal (reading span), visuospatial (symmetry span), and numeric (digit span) content. This feature allowed us to measure WM capacity as a domain-general construct (Oberauer et al., 2000; Oswald et al., 2015). The correlations between the implemented WM tasks were positive and greater than .40 for the Portuguese sample, and positive, though lower in magnitude, for the Brazilian sample (between .26 and .34; see Table 3). Redick et al. (2012) reported positive correlations between reading span and symmetry span ranging from .36 to .59, similar in magnitude to those observed in the present study (.40–.46). Additionally, Gonthier et al. (2016) reported correlations between reading span and symmetry span of similar magnitude (*r* = .33). Monteiro et al. (2024), in a sample of Portuguese participants, observed correlations between reading and symmetry span (*r* = .52) that are in a similar ballpark to the observed here. Digit span also correlated moderately with the complex span tasks. Kane et al. (2004) observed that digit span correlated at *r* = .59 and *r* = .44 with reading and symmetry span, which correlated to each other at *r* = .60. These results suggest that simple span measures such as digit span are similarly predictive of WM capacity as complex span measures. Colom et al. (2006) reanalyzed several data sets that included simple and complex span measures and observed that both types of tasks were similarly predictive of high cognition. Our findings are in line with this proposition: measures of WM capacity may include different sets of tasks and demands, and this variability may be beneficial for increasing its predictive validity. Accordingly, Monteiro et al. (2024) demonstrated via permutation analyses that WM latent factors derived from heterogeneous tasks have higher predictive value for measures of intelligence. The inclusion of three tasks with different materials and paradigms is therefore a plus factor of the battery, and future users should include all tasks when measuring WM capacity.

### Sample Differences

In general, the task scores from the Brazilian sample were lower than those from the European Portuguese sample. There are many factors that could contribute to explaining these differences. First, they may be attributed to contextual factors during data collection. Specifically, some participants in Brazil completed the battery in a classroom setting, which included the presence of a teacher and classmates, potentially introducing distractions. Furthermore, in the classroom environment, participants lacked the ability to select the environment conditions to do the task, such as selecting a quiet space or optimizing perceived attention capacity. Second, the social economic status of the participants in Brazil and Portugal may differ. Although the Brazilian sample also included university students, we cannot ascertain that they were in comparable socioeconomic conditions. According to the last Human Development Index report (United Nations, 2023/2024), Portugal is in the 42° position in the world ranking, whereas Brazil figures in the 89° position. This index takes into consideration income, education, and life expectancy. It is worth noting that this project was conducted amidst the Covid-19 pandemic (data collection occurred in the second semester of 2021), which had a more substantial impact in Brazil than Portugal in terms of mental health (Vitorino et al., 2022). Although we did not observe a significant difference between performance of participants that reported having contracted Covid-19 compared to the ones that were not diagnosed with Covid-19, the number of participants that contracted Covid-19 was substantially larger in Brazil than in Portugal. These results are in line with the possibility that the impact of the pandemic was larger in Brazil, and the overall situation of the pandemic could have placed a larger cognitive burden on these participants.

Despite differences in the overall level of performance between the samples, tasks scores correlated similarly within each sample and their shared variance was captured by a single factor reflecting WM capacity. Accordingly, we obtained configural and metric invariance in the CFA multigroup analysis. Unfortunately, scalar invariance was not observed. This means that the latent means of these two groups cannot be directly compared (Chen, 2008; Horn & Mcardle, 1992). Future studies that aim to compare the performance of Portuguese and Brazilian samples will need to first examine whether they can observe scalar invariance in the test scores.

### Speed-Accuracy Tradeoffs

The tendency to sacrifice performance on the processing task – being faster but less accurate – in order to achieve better performance on the memory component of the complex task, and vice versa, was not observed in this study. Instead, the correlations between the processing and memory components of the two complex span tasks were positive and significant. Therefore, we did not impose any minimum performance level on the processing component, which allowed us to maintain the data of the whole sample.

The battery instructions indicated a limited time for the processing task and emphasized accuracy on the memory component. The use of computer-paced processing demands is recommended in complex pan tasks, as it generally increases its predictive value (McCabe, 2010). In lab.js, it is straightforward to adjust instructions or introduce parameters to experimentally examine variables of interest related to speed-accuracy tradeoffs. For instance, this could involve manipulating the instructions themselves, altering incentives, or setting prompts to request participant responses at specific moments in time (Glickman et al., 2005).

## Conclusion

In sum, the study presents a computerized battery for WM assessment that is suitable for online use in Portugal and Brazil and demonstrates good internal reliability. A confirmatory factor analysis indicated that the implemented tasks loaded onto a single factor, reflecting WM capacity. This instrument enables intercultural studies and research on WM by providing a method for collecting data across different countries. Its implementation using lab.js offers a flexible and customizable format that can be utilized in both laboratory and online settings. The findings align with data patterns observed in other studies, suggesting the battery’s convergent validity. The battery is applicable in the field of experimental psychology and in research exploring individual differences in cognitive abilities. Future research should aim to collect data from a broader age range of participants in Portugal and Brazil and to generate norms that enable comparisons between individual participants and the general population. Once validated, the battery can also be tested in English-speaking countries.

## Data Accessibility Statement

All data, materials, and analysis scripts related to the research reported in this article are publicly available at https://osf.io/ubcez.

## Footnotes

We included only a version of forward span which requires the reproduction of a sequence of digits in the same order as they were presented. Backward digit span – which requires the reproduction of the sequence in reverse order – is often considered as also assessing the information-processing function. Backward digit span would, however, be very straightforward to implement in our battery. The full task code is available and could be readily adapted for this purpose.
